# Supplementary material for: Contaminant occurrence, distribution and ecological risk assessment of phthalate esters in the Persian Gulf
Source: PLoS One. 2023 Jul 7;18(7):e0287504. doi: 10.1371/journal.pone.0287504 (PMC10328224; doi:10.1371/journal.pone.0287504)
Supplement: S1 Table — (DOCX) [file pone.0287504.s001.docx]

Contaminant occurrence, distribution and ecological risk assessment of phthalate esters in the Persian Gulf

Maria Khishdost ^a, b^, Sina Dobaradaran^c,d,e^, Gholamreza Goudarzi^a,b,f^, Afshin Takdastan^a,b^, Ali Akbar Babaei^a,b*^

^a^ Department of Environmental Health Engineering, School of Public Health, Ahvaz Jundishapur University of Medical Sciences, Ahvaz, Iran

^b^ Environmental Technologies Research Center, Ahvaz Jundishapur University of Medical Sciences, Ahvaz, Iran

^c^ Systems Environmental Health and Energy Research Center, The Persian Gulf Biomedical Sciences Research Institute, Bushehr University of Medical Sciences, Bushehr, Iran

^d^ Department of Environmental Health Engineering, Faculty of Health and Nutrition, Bushehr University of Medical Sciences, Bushehr, Iran

^e^ Instrumental Analytical Chemistry and Centre for Water and Environmental Research (ZWU), Faculty of Chemistry, University of Duisburg-Essen, Universitätsstr. 5, Essen, Germany

^f^ Air Pollution and Respiratory Diseases Research Center, Ahvaz Jundishapur University of Medical Sciences, Ahvaz, Iran

^*^Corresponding author at: Environmental Technologies Research Center, Ahvaz Jundishapur University of Medical Sciences, Ahvaz, Iran. E-mail address: [ababaei52@gmail.com](mailto:ababaei52@gmail.com)

**Table S1.** Comparison of PAEs concentrations in global water resources (μg/L)

| Location | DMP | DEP | DIBP | DBP | DEHP | DOP | BBP | Reference |
| --- | --- | --- | --- | --- | --- | --- | --- | --- |
| Persian Gulf | ND-0.1 | ND-0.2 | 0.2-3.1 | ND-0.4 | 5.7-18.5 | 0.2-4.6 | ND | This study |
| northern South China Sea | 0.00174–0.0136 | <MQL–0.0323 | <MQL–0.0348 | <MQL–0.358 | 0.0127–1.05 | ND | - | (1) |
| Jiulong River, China | 0.03–0.24 | 0.01–0.09 | 1.09–11.8 | 0.03–1.77 | 0.62–12.43 | - | - | (2) |
| Northwest Pacific marginal seas | <LOQ–0.0045 | 0.0013–0.0702 | 0.0050–0.0181 | 0.0112–0.0434 | 0.0250–0.3407 | <LOQ | - | (3) |
| U-Tapao Canal, Southern, Thailand | - | - | 2.12 | 1.87 | 2.51 | - | - | (4) |
| Songhua River, China | 0.98–4.12 | 1.33–6.67 | - | 1.69–11.8 | 2.26–11.6 | - | - | (5) |
| Yangtze River, China | 0.0006–0.0322 | 0.0003–0.011 | 0.0805–1.468 | 0.183–3.373 | 0.125–4.097 | ND–0.0246 | ND | (6) |
| Bottled water, Tehran | 0.84 | 0.5 | - | 0.5 | 2.22 | 1.12 | 0.93 | (7) |

<MQL: Below method quantification limits; <LOQ: Below limit of quantification; ND: Not detected.

1. Cao Y, Li J, Wu R, Lin H, Lao J-Y, Ruan Y, et al. Phthalate esters in seawater and sediment of the northern South China Sea: Occurrence, distribution, and ecological risks. Science of the Total Environment. 2022;811:151412.

2. Li R, Liang J, Gong Z, Zhang N, Duan H. Occurrence, spatial distribution, historical trend and ecological risk of phthalate esters in the Jiulong River, Southeast China. Science of the Total Environment. 2017;580:388-97.

3. Paluselli A, Kim S-K. Horizontal and vertical distribution of phthalates acid ester (PAEs) in seawater and sediment of East China Sea and Korean South Sea: Traces of plastic debris? Marine pollution bulletin. 2020;151:110831.

4. Kingsley O, Witthayawirasak B. Occurrence, ecological and health risk assessment of phthalate esters in surface water of U-Tapao Canal, southern, Thailand. Toxics. 2020;8(3):58.

5. Gao D, Li Z, Wen Z, Ren N. Occurrence and fate of phthalate esters in full-scale domestic wastewater treatment plants and their impact on receiving waters along the Songhua River in China. Chemosphere. 2014;95:24-32.

6. Weizhen Z, Xiaowei Z, Peng G, Ning W, Zini L, Jian H, et al. Distribution and risk assessment of phthalates in water and sediment of the Pearl River Delta. Environmental Science and Pollution Research. 2020;27(11):12550-65.

7. Mehraie A, Shariatifar N, Arabameri M, Moazzen M, Mortazavian AM, Sheikh F, et al. Determination of phthalate acid esters (PAEs) in bottled water distributed in tehran: A health risk assessment study. International Journal of Environmental Analytical Chemistry. 2022:1-15.
