# Supplementary figures and images for: Contaminant occurrence, distribution and ecological risk assessment of phthalate esters in the Persian Gulf
Source: PLoS One. 2023 Jul 7;18(7):e0287504. doi: 10.1371/journal.pone.0287504 (PMC10328224; doi:10.1371/journal.pone.0287504)

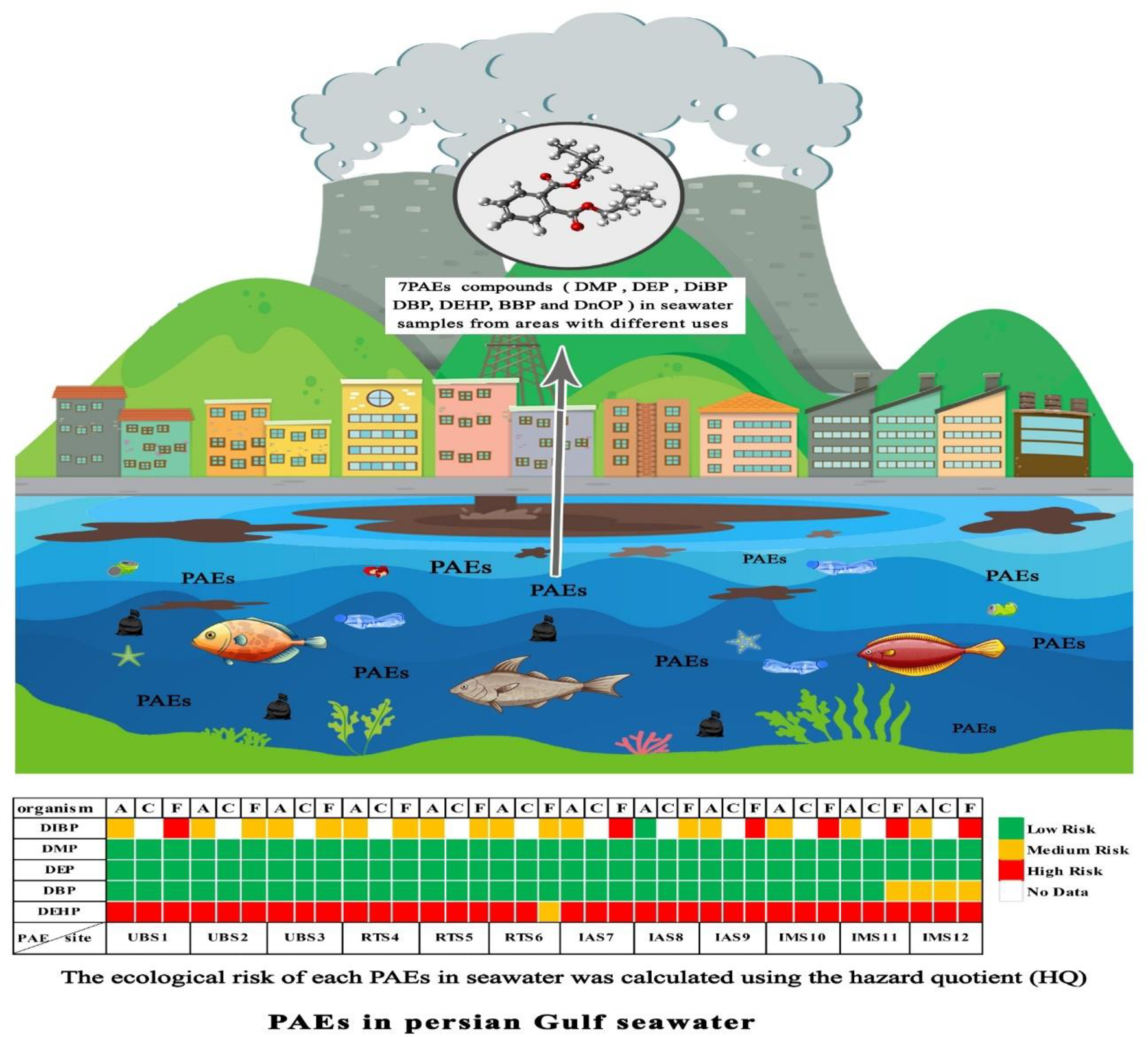

Supplement: S1 Graphical abstract — (TIF) [file pone.0287504.s002.tif]
