# Supplementary material for: Contaminant occurrence, distribution and ecological risk assessment of phthalate esters in the Persian Gulf
Source: PLoS One. 2023 Jul 7;18(7):e0287504. doi: 10.1371/journal.pone.0287504 (PMC10328224; doi:10.1371/journal.pone.0287504)
Supplement: Attachment — Submitted filename: Response to Reviewers-2 (1).docx [file pone.0287504.s003.docx]

Ms. No.: PONE-D-23-03668

Contaminant occurrence, distribution and ecological risk assessment of phthalate esters in the Persian Gulf

**Dear Prof. Hai O. Xu,**

Editor - PLOS ONE,

Please find below the detailed responses to the Reviewer’s/Editor’s comments. We have also enclosed the thoroughly revised version of the manuscript mentioned above according to the Reviewer’s comments, suggestions, and recommendations. The changes made have been highlighted with **yellow background** in color.

**Editor’s and Reviewer’s comments:**

**Editor's comments:**

1. Please ensure that your manuscript meets PLOS ONE's style requirements, including those for file naming. The PLOS ONE style templates can be found at

https://journals.plos.org/plosone/s/file?id=wjVg/PLOSOne_formatting_sample_main_body.pdf and

<https://journals.plos.org/plosone/s/file?id=ba62/PLOSOne_formatting_sample_title_authors_affiliations.pdf>

Thank you a lot, for your advice. We checked it.

2. In your Methods section, please provide additional information regarding the permits you obtained for the work. Please ensure you have included the full name of the authority that approved the field site access and, if no permits were required, a brief statement explaining why.

Field site access was granted by the Head Office of the Bushehr Province Environmental. We added this to the Method section.

3. Thank you for stating the following in the Acknowledgments Section of your manuscript:

"This study is the result of the Ph.D. thesis in Environmental Health Engineering of Ahvaz Jundishapur University of Medical Sciences of Medical Sciences (No. ETRC‒9901)."

We note that you have provided funding information that is not currently declared in your Funding Statement. However, funding information should not appear in the Acknowledgments section or other areas of your manuscript. We will only publish funding information present in the Funding Statement section of the online submission form.

Please remove any funding-related text from the manuscript and let us know how you would like to update your Funding Statement. Currently, your Funding Statement reads as follows:

"We thank Ahvaz Jundishapur University of Medical Sciences because the study received ethical clearance (IR.AJUMS.REC.1399.070) from the Ethics Committee of the Ahvaz Jundishapur University of Medical Sciences (Iran) under project No. ETRC‒9901. The Ahvaz Jundishapur University of Medical Sciences had no role in the study design, data collection, and analysis, the decision to publish, or the preparation of the manuscript."

Please include your amended statements within your cover letter; we will change the online submission form on your behalf.

Thanks for your nice overview. We removed the part Acknowledgments Section.

4. We note that you have indicated that data from this study are available upon request. PLOS only allows data to be available upon request if there are legal or ethical restrictions on sharing data publicly. For more information on unacceptable data access restrictions, please see http://journals.plos.org/plosone/s/data-availability#loc-unacceptable-data-access-restrictions.

In your revised cover letter, please address the following prompts:

a) If there are ethical or legal restrictions on sharing a de-identified data set, please explain them in detail (e.g., data contain potentially sensitive information, data are owned by a third-party organization, etc.) and who has imposed them (e.g., an ethics committee). Please also provide contact information for a data access committee, ethics committee, or other institutional body to which data requests may be sent.

b) If there are no restrictions, please upload the minimal anonymized data set necessary to replicate your study findings as either Supporting Information files or to a stable, public repository and provide us with the relevant URLs, DOIs, or accession numbers. For a list of acceptable repositories, please see http://journals.plos.org/plosone/s/data-availability#loc-recommended-repositories.

We will update your Data Availability statement on your behalf to reflect the information you provide.

This part was edited as “All relevant data are within the manuscript.”

5. PLOS requires an ORCID iD for the corresponding author in Editorial Manager on papers submitted after December 6th, 2016. Please ensure that you have an ORCID iD and that it is validated in Editorial Manager. To do this, go to ‘Update my Information’ (in the upper left-hand corner of the main menu), and click on the Fetch/Validate link next to the ORCID field. This will take you to the ORCID site and allow you to create a new iD or authenticate a pre-existing iD in Editorial Manager. Please see the following video for instructions on linking an ORCID iD to your Editorial Manager account: <https://www.youtube.com/watch?v=_xcclfuvtxQ>

Many thanks, it was done in title page.

6. Your ethics statement should only appear in the Methods section of your manuscript. If your ethics statement is written in any section besides the Methods, please move it to the Methods section and delete it from any other section. Please ensure that your ethics statement is included in your manuscript, as the ethics statement entered into the online submission form will not be published alongside your manuscript.

Many thanks, it was done.

7. We note that Figures 1 and 3 in your submission contain [map/satellite] images which may be copyrighted. All PLOS content is published under the Creative Commons Attribution License (CC BY 4.0), which means that the manuscript, images, and Supporting Information files will be freely available online, and any third party is permitted to access, download, copy, distribute, and use these materials in any way, even commercially, with proper attribution. For these reasons, we cannot publish previously copyrighted maps or satellite images created using proprietary data, such as Google software (Google Maps, Street View, and Earth). For more information, see our copyright guidelines: http://journals.plos.org/plosone/s/licenses-and-copyright.
 We require you to either (a) present written permission from the copyright holder to publish these figures specifically under the CC BY 4.0 license, or (b) remove the figures from your submission:
 a. You may seek permission from the original copyright holder of Figures 1 and 3 to publish the content specifically under the CC BY 4.0 license.  
 We recommend that you contact the original copyright holder with the Content Permission Form (http://journals.plos.org/plosone/s/file?id=7c09/content-permission-form.pdf) and the following text:
“I request permission for the open-access journal PLOS ONE to publish XXX under the Creative Commons Attribution License (CCAL) CC BY 4.0 (http://creativecommons.org/licenses/by/4.0/). Please be aware that this license allows unrestricted use and distribution, even commercially, by third parties. Please reply and provide explicit written permission to publish XXX under a CC BY license and complete the attached form.”
 Please upload the completed Content Permission Form or other proof of granted permissions as an "Other" file with your submission.
 In the figure caption of the copyrighted figure, please include the following text: “Reprinted from [ref] under a CC BY license, with permission from [name of publisher], original copyright [original copyright year].”
 b. If you are unable to obtain permission from the original copyright holder to publish these figures under the CC BY 4.0 license or if the copyright holder’s requirements are incompatible with the CC BY 4.0 license, please either i) remove the figure or ii) supply a replacement figure that complies with the CC BY 4.0 license. Please check copyright information on all replacement figures and update the figure caption with source information. If applicable, please specify in the figure caption text when a figure is similar but not identical to the original image and is therefore for illustrative purposes only.
The following resources for replacing copyrighted map figures may be helpful:
 USGS National Map Viewer (public domain): http://viewer.nationalmap.gov/viewer/
The Gateway to Astronaut Photography of Earth (public domain): http://eol.jsc.nasa.gov/sseop/clickmap/
Maps at the CIA (public domain): https://www.cia.gov/library/publications/the-world-factbook/index.html and https://www.cia.gov/library/publications/cia-maps-publications/index.html
NASA Earth Observatory (public domain): http://earthobservatory.nasa.gov/
Landsat: http://landsat.visibleearth.nasa.gov/
USGS EROS (Earth Resources Observatory and Science (EROS) Center) (public domain): http://eros.usgs.gov/#
Natural Earth (public domain): <http://www.naturalearthdata.com/>

Thanks for your attention, the figs changed, we used the <https://data.humdata.org/organization/ocha-romena> site

**Reviewers’ comments:**

**Reviewer #1:**

1. - The paper has some typographical and grammatical errors which must be corrected. Therefore, the English language of the text should be strongly revised by a native English speaker with expertise in the scientific field and skills in scientific paper writing.

Thank you for your valuable comment. The entire text of the article was checked for the English language.

2. -The "conclusion" section in the abstract part must be improved.

Thanks for your nice overview and useful comments. The whole abstract has been revised and updated.

3. - The novelty is not sufficiently explained or clears in the introduction section. Also, the researches gap should be clearly described.

It was provided, Line 139-141.

4. - The quality control/assurance and the analytical recovery data from PAEs in seawater samples must be presented.

Thanks for your attention, it was provided, Line 197- 201and Table.2

5. - Calibration ranges for the considered analytes, as well as at least one chromatogram illustrating the separation of standard PAEs + separation of PAEs in a seawater sample must be provided.

Thanks for your attention, Fig 2 Added.

6. - The results of the 'One sample T test' must be presented for comparison of the mean contents of in seawater samples with maximum permissible concentration.

Thank you very much for your excellent suggestion. Your concern has been added to the statistics analysis and results section.(line 219-222 &258-272).

7. - Quality of the discussion section must be improved. In so doing, the authors must be organized the discussion from the general to the specific, linking your findings to the literature, then to theory, then practice and avoid repetition from the introduction.

Thank you for your comment. The discussion has been edited according to your valuable suggestion

8. - Limitations of the study must be presented in the conclusion section.

Thank you for your valuable comment, below the conclusion, the limitation and strength section was added

9. - For numbers in text and tables < 1.00, use three digits beyond the decimal point; for numbers between 1.00 and 9.99 use two digits beyond the decimal point; for numbers between 10.0 and 99.9, use one digit beyond the decimal point; and for concentrations ≥ 100, use the nearest whole number.

Many thanks, it was provided in whole manuscript.

10. - The following relevant reference must be cited for in-text citation: Environmental Science and Pollution Research, 28(43): 61151-61162 (2021).

Thank you for sharing this valuable study, we used it for improving the current manuscript.

**Reviewer #2:**

It is a good and practical study but I made the following observations which should be addressed by the authors. After doing the following points, it can be accepted in the Journal of PLOS ONE.

Thanks for your nice overview and finding the work suitable to warrant publication.

**Abstract**.

1. - It needs to be rewritten. The abstract should be improved, very briefly the purpose of the study, methodology, results and conclusions should be mention.

Thanks for your useful comments. The whole abstract has been revised and updated.

**Keywords**

2. - Sort by alphabetical order.

Keywords has been revised and updated

3. - It is better to use keywords other than the title.

Keywords has been revised and updated

**Introduction**

4. - It is necessary to give explanations about the preparation method and equipment such as GC-MS, etc.

Thanks for your useful comments. Added to introduction section (line 90-99).

5. - It is necessary to explain a little about the risk assessment.

Thank you very much for your valuable comment. It was provided. lines,115-21.

6. - In the introduction section, it is necessary to mention the limitations of the previous studies (briefly) and explain the reason for the current study in more detail. It is also necessary to briefly mention the benefits of doing this research.

Many thanks, it was done. Lines 123-29

7. - You can use the following articles to complete this section:
   https://doi.org/10.1007/s11356-023-25313-0, https://doi.org/10.1080/03067319.2022.2062239, https://doi.org/10.1007/s11356-018-1471-y, https://doi.org/10.1007/s11356-021-14290-x,

Thank you so much, we used the valuable proposed related references to improve this section.

**Materials and methods**

8. - Page 5, Line 109. Change to “(C₁₀H₁₀O₄ -Purity ≥99%),”

Many thanks, it was done.

9. - Page 5, Line 113. Change to “(H₂SO₄ -Purity ≥95%),”

Thank you, it was done.

10. - The space between numbers and units should be observed throughout the text (such as Line 135. “1 L”, etc.)

Many thanks, it was done.

11. - Line 140. It is necessary to add a reference or a summary of it to the magnetic absorber section.

It was added.

12. - It is better to move the “Data analysis” part to the end of the materials and methods section.

Many thanks, it was moved.

13. - It is necessary to mention the preparation steps of the standard and internal standard in detail.

Thank you for your valuable comment. Your concern has been added to the Sample preparation and analyses. (Line 190-194)

14. - It is necessary to mention the LOD, LOQ, Recovery and QC samples.

Thanks for your attention, it was provided, Line 197- 201 and Table.2

**Result and Discussion**

15. - The discussion part of the article is extremely weak; it is necessary to mention possible reasons for all the results and to compare with other works. Such as Part 3.1., 3.2., 3.3. and 3.4. Should be written in more detail (more comparisons should be made with past studies (at least 7 similar studies, even with other similar food matrix such as bottled water, etc.) and possible reasons for the higher or lower values of these compounds should be mentioned). You can use the mentioned articles.

Thank you for your comment. The discussion has been edited according to your valuable suggestion

16. - In Table 2, the maximum and minimum of each data should also be mentioned.

It was mentioned based on your comment.

Conclusion

17. -Finally, state your limitations of the study and possible suggestions for other researchers.

Thank you very much for your valuable comment, added limitation and possible suggestion.

**Reviewer #3:**

（1） line 75: please specify whether the data about the PAEs is Persian Gulf or global.

It is globally and specified in manuscript.

（2） Please supplement the existing research progress and data on PAEs.

Thank you so much. This table was transferred to the supplemental file.

（3） Line 184: Data on PAEs in the area before previous industrial, agricultural and domestic wastewater discharges are lacking, so it cannot be directly concluded that high concentrations of PAEs in the area originate from them. The specific type of industries in its vicinity should be given, not all of them can lead to PAEs pollution

Thanks for your attention, it was checked and modified based on this comment. Line, 241-42 and 260-76.

（4） Line 187: Please give a specific value, how high is it?

It was done, Line 245.

（5） Line 188: please check the correctness of writing “PAES”.

Thank you for your attention. It was corrected in whole parts of manuscript.

（6） Line 209-Line 213: From the results and geographical location, the concentrations of Σ6PAEs in Bushehr urban site is higher than that in Asaloye Industrial site. Give a reasonable explanation for this.

The purpose of this section is to emphasize the high concentration of PAEs in urban and industrial areas compared to rural areas. The cause of this may be the presence of PAEs in the released wastewater and also discarded waste, as well as the further development of tourism in urban areas.

The difference between urban and industrial areas is not significant, which indicates the impact of human activities on pollutant emission. it was mentioned in Line 283-292.

（7） Line 234: please check the correctness of writing “Y angtze”

Thank you for your attention. It was corrected. Line 304 and 307.
